# Outcomes in Asymptomatic Patients Undergoing Coronary Revascularization Before Liver Transplantation

**DOI:** 10.3390/jcm14197067

**Published:** 2025-10-07

**Authors:** Goyal Umadat, Jennifer Lee, Jordan C. Ray, Ryan M. Chadha, Yaohua Ma, Hanna J. Sledge, Surakit Pungpapong, Leslie E. Janik, Dilip Pillai, Abdallah El Sabbagh, Peter Pollak, Shahyar M. Gharacholou

**Affiliations:** 1 Division of Cardiovascular Diseases, Mayo Clinic Florida, Jacksonville, FL 32224, USA; ray.jordan@mayo.edu (J.C.R.); janik.leslie@mayo.edu (L.E.J.); pillai.dilip@mayo.edu (D.P.); elsabbagh.abdallah@mayo.edu (A.E.S.); pollak.peter@mayo.edu (P.P.); gharacholou.shahyar@mayo.edu (S.M.G.); 2 Division of Anesthesiology and Perioperative Medicine, Mayo Clinic, Rochester, MN 55902, USA; lee.jennifer3@mayo.edu; 3 Division of Anesthesiology and Perioperative Medicine, Mayo Clinic Florida, Jacksonville, FL 32224, USA; chadha.ryan@mayo.edu; 4 Division of Clinical Trials and Biostatistics, Mayo Clinic Florida, Jacksonville, FL 32224, USA; ma.yaohua@mayo.edu (Y.M.); sledge.hanna@mayo.edu (H.J.S.); 5 Division of Gastroenterology and Hepatology, Mayo Clinic Florida, Jacksonville, FL 32224, USA; pungpapong.surakit@mayo.edu

**Keywords:** liver transplant, coronary artery disease, revascularization

## Abstract

**Background:** Coronary artery disease (CAD) is common among liver transplantation (LT) candidates, yet whether pre-transplant percutaneous coronary intervention (PCI) improves post-LT outcomes remains uncertain. **Methods:** We conducted a single-center, Institutional Review Board-approved cohort study of adults undergoing LT from 2005 to 2025. Asymptomatic candidates with significant stenosis on invasive angiography were included; prior coronary artery bypass grafting was excluded. The primary endpoint was major adverse cardiovascular events (MACE: myocardial infarction [MI], stroke/transient ischemic attack, new systolic dysfunction, post-LT coronary revascularization, or all-cause death). **Results:** Among 111 patients (median age 65 years; 84% male), 66 (59%) underwent PCI and 45 (41%) were managed medically. Over a median 32 months of follow-up, 61 patients (55%) experienced MACE. Composite MACE did not differ between PCI and non-PCI groups (52% vs. 60%, *p* = 0.40; log-rank *p* = 0.59). Fine–Gray modeling showed no association of PCI with MACE; independent predictors were prior MI (HR 1.81, 95% CI 1.01–3.24) and pre-transplant dialysis (HR 2.13, 95% CI 1.07–4.24). Major bleeding occurred in 7%. Matched and era-stratified analyses were concordant. **Conclusions:** In asymptomatic LT candidates with angiographically severe CAD, pre-LT PCI was not associated with a lower incidence of post-LT MACE.

## 1. Introduction

Coronary artery disease (CAD) is a well-recognized comorbidity among patients undergoing liver transplantation (LT). Cirrhosis contributes to a pro-inflammatory and pro-thrombotic state, predisposing individuals to atherosclerosis and CAD [1,2]. This risk is further amplified in patients with comorbid conditions such as hypertension, hyperlipidemia, and diabetes. The presence of CAD has significant implications for LT eligibility and has been associated with significant morbidity and mortality following liver transplantation [3,4,5,6,7,8]. Despite limited evidence from studies, many institutions have implemented routine cardiovascular risk assessments, including CAD screening and ischemic evaluations, in the pre-LT setting. The 2022 American College of Cardiology (ACC)/American Heart Association (AHA) scientific statement on CAD screening in LT candidates recommends that nearly all patients considered for transplantation undergo evaluation for CAD, specifically advocating for anatomic assessment via coronary computed tomographic angiography (CCTA) or invasive angiography [9]. However, the ACC/AHA guidelines on myocardial revascularization underscore the lack of data in LT candidates without an indication for intervention [10]. Significant CAD, once identified on anatomic testing, presents a challenging situation for LT centers as it is currently a 2C recommendation for coronary revascularization (CR), despite limited data its role in asymptomatic patients. Therefore, we studied post-LT major adverse cardiovascular events (MACE) among patients with significant CAD, who underwent CR via percutaneous coronary intervention (PCI) prior to their transplant.

## 2. Materials and Methods

This single-center, Institutional Review Board-approved study reviewed adults who underwent liver transplantation (LT) between January 1995 and April 2025. Although the cohort spans this entire interval, angiographic imaging data were available in the electronic medical record beginning in January 2005. Analyses requiring coronary angiography therefore relied on data from 2005 onward. Baseline characteristics included sex, race, age at transplant, obesity, etiology of end-stage liver disease, hypertension, hyperlipidemia, diabetes mellitus, history of transient ischemic attack (TIA)/stroke, prior myocardial infarction (MI), smoking history, and end-stage renal disease on dialysis.

Patients were eligible if they had asymptomatic, significant coronary stenosis on invasive coronary angiography pre-LT. Most underwent angiography after abnormal pre-LT stress testing; others were referred directly on the basis of high-risk clinical or imaging features (e.g., prior MI, substantial coronary artery calcification on coronary CT angiography, or metabolic syndrome). Revascularization decisions were made case-by-case by a multidisciplinary committee. Initially, the committee favored revascularization when one or two discrete lesions were readily amenable to percutaneous coronary intervention (PCI). When coronary anatomy was technically complex (e.g., risk of jailing a large side branch, Medina 1,1,1 lesion, long atretic segments, tortuous coronary anatomy, etc.), an expanded multidisciplinary discussion with interventional cardiology and cardiothoracic surgery determined the optimal strategy. When PCI was selected, all major epicardial target lesions were revascularized. Significant stenosis was defined as ≥70% diameter reduction on angiography (≥50% for the left main) or, when noninvasive functional testing was normal, physiologic ischemia by instantaneous wave-free ratio ≤0.89 or fractional flow reserve (FFR) ≤0.80. Patients considered for PCI completed a two-week dual antiplatelet therapy (DAPT) tolerance run-in with clopidogrel and low-dose aspirin before PCI, followed by three months of DAPT prior to listing. All PCI cases used drug-eluting stents confirmed by chart review. Patients who underwent coronary artery bypass grafting before LT were excluded.

Post-LT outcomes included MI, stroke/TIA, new systolic dysfunction, post-LT coronary revascularization with PCI, and all-cause mortality. MI followed the Fourth Universal Definition, requiring a rise and fall in high-sensitivity troponin with at least one value above the 99th percentile plus ischemic symptoms, ECG changes, or imaging evidence consistent with ischemia [11]. MI subtypes (type 1 vs. type 2) were adjudicated by manual chart review and inspection of coronary angiography. Patients meeting MI criteria underwent angiography to assess for intracoronary plaque rupture; in the absence of an acute culprit lesion, events were classified as type 2 MI. Stroke/TIA required a new imaging abnormality on brain CT or MRI as determined by a neurologist. New systolic dysfunction was defined as a left ventricular ejection fraction <50% compared with pre-transplant echocardiography, or an absolute decrease of ≥10% if the pre-transplant ejection fraction was already <50%. The primary composite endpoint (MACE) comprised MI, stroke/TIA, new-onset systolic dysfunction, post-LT coronary revascularization, or death from any cause. Pre-transplant PCI was the exposure of interest, and time-to-event analyses were anchored at the date of transplantation. Given bleeding concerns after PCI in the LT population, post-transplant bleeding served as a safety endpoint and was defined using Bleeding Academic Research Consortium (BARC) type 3 or 5 criteria—overt bleeding with hemoglobin drop ≥3 g/dL, any transfusion with overt bleeding, intracranial hemorrhage (excluding microbleeds or hemorrhagic transformation), or probable/definite fatal bleeding [12].

To mitigate treatment-selection bias when comparing revascularization with medical therapy, we constructed a matched cohort using coarsened exact matching after alternative matching approaches yielded suboptimal balance. Matching variables were age at transplant, gender, significant left anterior descending (LAD) artery disease, significant branch-vessel disease, prior MI, prior stroke, and dialysis dependence. We then estimated the association between pre-LT revascularization and MACE using cumulative incidence functions and Fine–Gray subdistribution hazard models with death as a competing event. Multivariable Fine–Gray models adjusted for prior stroke, prior MI, hypertension, dialysis dependence, age, diabetes, hyperlipidemia, metabolic dysfunction-associated steatotic liver disease (MASLD), male gender, current smoking, and pre-LT revascularization. Because follow-up spanned approximately two decades and stent technologies evolved during this period, we prespecified an era analysis stratified as 2005–2015 versus 2016–2025.

Continuous variables were summarized as medians with interquartile ranges and categorical variables as counts with percentages. Between-group comparisons used Kruskal–Wallis tests for continuous measures and Pearson χ^2^ tests for categorical measures to assess associations with coronary revascularization status. To address the study aims, hazard risk analysis was performed using univariate and multivariate Cox proportional hazards models, with hazard ratios and 95% confidence intervals estimated via log-rank tests. Kaplan–Meier survival curves were generated for the composite MACE endpoint as well as each individual component. All statistical tests were two-sided with α < 0.05, and analyses were conducted in R (version 4.2.2; R Foundation for Statistical Computing, Vienna, Austria).

## 3. Results

Between January 2005 and April 2025, 800 liver-transplant (LT) candidates underwent invasive coronary angiography; 122 (15%) had angiographically significant coronary artery disease (CAD). Eleven patients underwent coronary artery bypass grafting pre-LT and were excluded, yielding a final cohort of 111. Median age was 65 years (IQR, 59–70); 84% were male and 88% were White. The median interval from angiography to LT was 7 months (IQR, 1–13). Of the 111 patients, 66 (59%) underwent pre-LT percutaneous coronary intervention (PCI) and 45 (41%) were managed medically.

Table 1 demonstrates statistically significant differences by revascularization status: obesity was more prevalent in the non-PCI group (62% vs. 41%, *p* = 0.027) and alcohol-related liver failure was more frequent (40% vs. 21%, *p* = 0.032), whereas cryptogenic cirrhosis was more common among PCI patients (48% vs. 24%, *p* = 0.011). Other measured covariates—including hypertension, hyperlipidemia, diabetes, smoking, and prior myocardial infarction (MI)—did not differ significantly.

**Figure 1:**
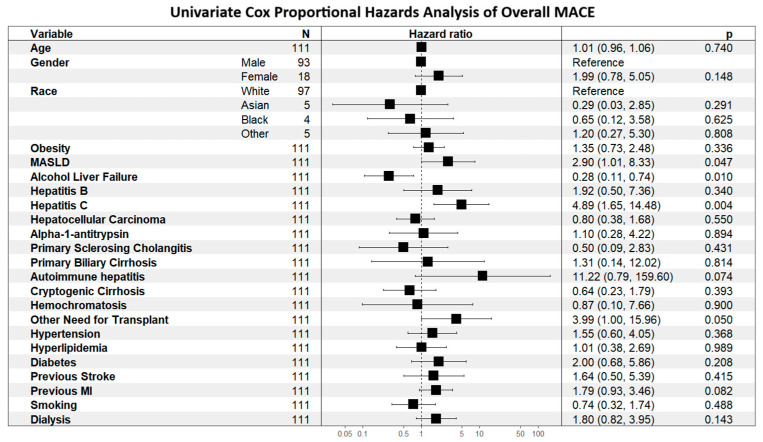
Univariate Cox Proportional Hazards Analysis of Overall MACE.

**Figure 2:**
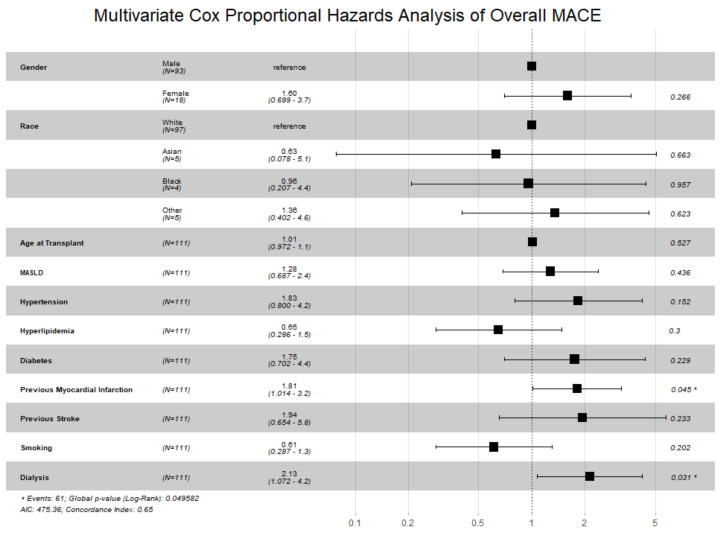
Multivariate Cox Proportional Hazards Analysis of Overall MACE. * = *p*-value < 0.05.

Determinants of MACE risk are shown first to establish context for outcome comparisons. As shown in Figure 1, alcohol-related liver disease was associated with lower MACE risk (HR 0.28, 95% CI 0.11–0.74), whereas hepatitis C was associated with higher risk (HR 4.89, 95% CI 1.65–14.48). As shown in Figure 2, prior MI (HR 1.81, 95% CI 1.01–3.24; *p* = 0.045) and pre-transplant dialysis (HR 2.13, 95% CI 1.07–4.24; *p* = 0.031) were independently associated with increased MACE risk.

During a median follow-up of 32 months (IQR, 13–74), 61 patients (55%) experienced ≥1 MACE. Table 2 reports event distributions: new-onset systolic dysfunction occurred in 28% of the cohort, followed by MI (23%), death (20%), stroke/TIA (6%), and post-LT coronary revascularization (6%). Twenty-six MIs occurred; 21 (81%) were type 2. Composite MACE incidence did not differ between PCI and non-PCI groups (52% vs. 60%, *p* = 0.40); mortality was 21% vs. 18% (*p* = 0.70). Major bleeding occurred in 8 patients (7%) without a significant difference by treatment strategy (9% vs. 4%, *p* = 0.47). Supplementary Table S1 provides incidence rates per 100 person-years: rates were similar for MI (4.33 vs. 5.14), stroke (0.98 vs. 1.31), revascularization (1.35 vs. 0.96), and systolic dysfunction (5.62 vs. 6.23). Overall MACE (15.2 vs. 11.1) and death (5.69 vs. 2.70) were numerically higher in PCI recipients.

Unadjusted associations between PCI and outcomes are provided in Table 3. PCI was not associated with the composite endpoint (HR 1.16, 95% CI 0.68–1.96; *p* = 0.60) or with MI (HR 0.73, 95% CI 0.33–1.61), stroke/TIA (HR 0.50, 95% CI 0.11–2.22), systolic dysfunction (HR 0.66, 95% CI 0.32–1.35), death (HR 1.73, 95% CI 0.68–4.37), or major bleeding (HR 1.87, 95% CI 0.38–9.34; *p* = 0.40).

**Figure 3:**
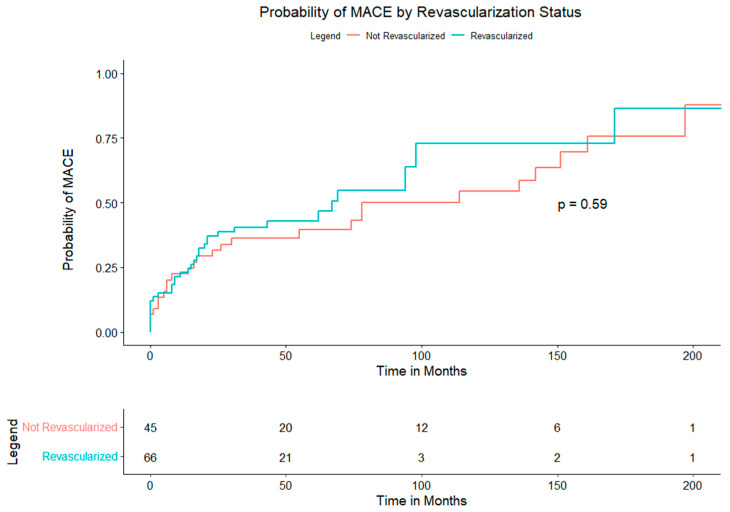
Probability of MACE by Revascularization Status.

Time-to-event analyses are shown in the Kaplan–Meier curves. Figure 3 (composite MACE) displays overlapping survival functions for PCI and non-PCI (log-rank *p* = 0.59); median event-free survival was 41 months with PCI and 38 months without PCI. Component end points similarly showed no between-group differences: MI (Supplementary Figure S1A, *p* = 0.44), stroke/TIA (Supplementary Figure S1B, *p* = 0.35), post-LT revascularization (Supplementary Figure S1C, *p* = 0.89), systolic dysfunction (Supplementary Figure S1D, *p* = 0.26), and death (Supplementary Figure S1E, *p* = 0.25). During the peri-operative period 11 (10%) patients experienced at least one MACE. Peri-operative MACE included 10 MIs (9%), 2 strokes (2%), 11 new diagnoses of systolic dysfunction (10%), and 5 deaths (5%). There were also 5 cases of major bleeding during the surgical period, representing 63% of all cases within the study cohort.

**Figure 4:**
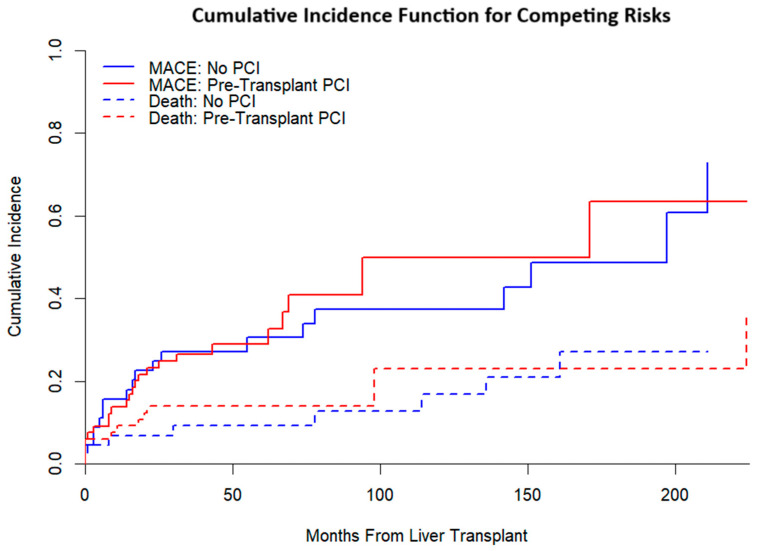
Cumulative Incidence Function for MACE with Death as a Competing Outcome.

**Figure 5:**
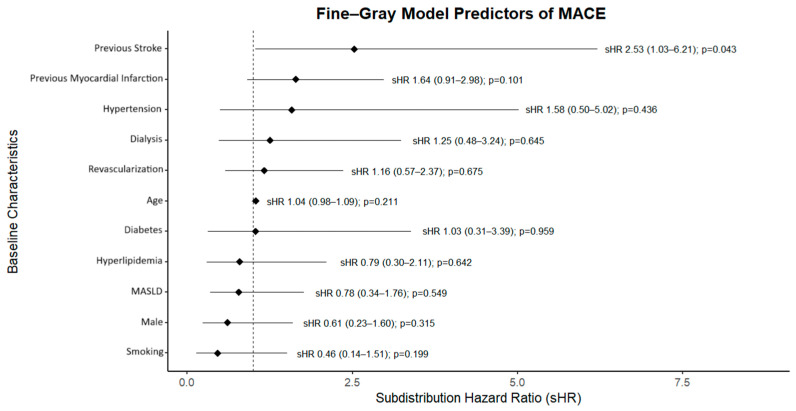
Fine-Gray Model of MACE with Death as the Competing Factor.

Competing-risks analysis (Figure 4) yielded broadly similar cumulative incidence functions for MACE by treatment group when accounting for death as a competing event. In the Fine–Gray model (Figure 5), pre-LT PCI was not associated with MACE (subdistribution HR 1.16, 95% CI 0.57–2.37; *p* = 0.675), whereas prior stroke was associated with higher risk (sHR 2.53, 95% CI 1.03–6.21; *p* = 0.043).

Effect measures from a binary perspective are reported in Table 4. Point estimates for MI (RR 0.58; OR 0.49), stroke (RR 0.51; OR 0.49), and systolic dysfunction (RR 0.64; OR 0.53) numerically favored PCI, whereas death (RR 1.19; OR 1.25) and major bleeding (RR 2.05; OR 2.15) numerically favored no PCI. All 95% confidence intervals encompassed the null; attributable risk estimates crossed zero.

The anatomical distribution and management of severe CAD are summarized in Table 5. Left anterior descending (LAD) lesions were more frequently revascularized (78% vs. 31%, *p* < 0.01), whereas branch-vessel disease was more often managed medically (49% vs. 29%, *p* = 0.04). No statistically significant differences in management were observed for left main, left circumflex, or right coronary artery disease. Vessel-specific outcomes (Table 6) showed no statistically significant associations between PCI and composite MACE or individual components across territories. LAD revascularization demonstrated a non-significant trend toward higher composite MACE (HR 1.60, 95% CI 0.94–2.71; *p* = 0.08).

Matched-cohort characteristics are provided in Table 7; covariate balance is visualized in the CEM love plot (Supplementary Figure S2), which showed standardized mean differences near zero post-matching (SMD < 0.10 threshold). Obesity remained more prevalent in the non-PCI group (69% vs. 22%, *p* = 0.01). Matched-cohort outcomes (Table 8) did not demonstrate statistically significant differences in composite MACE (HR 0.33, 95% CI 0.08–1.33; *p* = 0.13) or component events; event counts were limited and confidence intervals were wide.

Era-stratified analyses (Table 9A,B) were consistent with the primary findings. From 2005 to 2015, no statistically significant associations between PCI and any outcome were noted (e.g., death HR 2.41, 95% CI 0.53–10.84). From 2016 to 2025, there was no association between PCI and composite MACE (HR 0.82, 95% CI 0.44–1.53; *p* = 0.54), MI (HR 0.62, 95% CI 0.25–1.59), or systolic dysfunction (HR 0.60, 95% CI 0.26–1.38). Supplementary Table S2 (Cohen’s *d*) showed uniformly small standardized effects (*d* ≤ 0.31) with confidence intervals including zero.

Across multivariable modeling, survival and competing-risk analyses, person-time incidence rates, vessel-specific assessments, matched analyses, and era-stratified evaluations, pre-LT PCI in asymptomatic LT candidates with angiographically severe CAD was not associated with a reduction in post-LT MACE.

## 4. Discussion

In this single-center cohort spanning two decades, pre-liver transplant percutaneous coronary intervention in asymptomatic candidates with angiographically severe coronary artery disease was not associated with a reduction in post-transplant major adverse cardiovascular events. Across multiple analytic approaches—including unadjusted survival analyses, competing-risks models, multivariable Fine–Gray regression, person-time incidence rates, vessel-specific analyses, coarsened exact matching, and era-stratified evaluations—PCI did not demonstrate a protective association with the composite endpoint of myocardial infarction, stroke or transient ischemic attack, new systolic dysfunction, post-LT coronary revascularization, or all-cause mortality. Event-free survival did not differ by treatment group, and cumulative incidence functions were broadly similar after accounting for death as a competing risk. LAD lesions were more frequently revascularized, yet there was a non-significant trend toward higher MACE in these patients, indicating that coronary territory alone may not be sufficient to guide revascularization decisions in this population. Instead, comorbid conditions—specifically prior myocardial infarction, dialysis dependence, and a history of stroke—were independently associated with increased post-transplant MACE, underscoring the prognostic importance of systemic risk factors.

These findings provide a counterpoint to earlier studies that reported improved cardiovascular outcomes in revascularized LT candidates. Prior analyses by Satapathy et al. and Wray et al. compared revascularized patients with CAD against those without angiographically significant disease and attributed observed differences to PCI [13]. Such comparisons are inherently confounded by differences in baseline cardiovascular risk between groups. By restricting the present analysis to patients with angiographically severe CAD, we provide a more direct assessment of the role of PCI in this population. The absence of measurable benefit parallels evidence from the Coronary Artery Revascularization Prophylaxis (CARP) trial in vascular surgery patients and subsequent meta-analyses in renal transplant candidates, both of which demonstrated no advantage to prophylactic revascularization in asymptomatic, stable CAD [14,15]. These findings reinforce concerns that PCI in LT candidates without symptoms may not improve long-term outcomes.

The 2013 AASLD practice guidance and the 2024 EASL Clinical Practice Guidelines acknowledge the limited and indirect evidence base supporting PCI in LT candidates [16,17]. The 2022 ACC/AHA scientific statement recommends CAD screening before LT but emphasizes that the benefit of revascularization in asymptomatic patients remains unproven. More recent consensus statements have stressed individualized decision-making, particularly given the bleeding risk associated with dual antiplatelet therapy in cirrhotic patients [18,19]. The absence of an outcome difference in this study is consistent with these recommendations.

Several limitations must be considered when interpreting the results of this study. The retrospective, single-center design introduces potential for selection bias and unmeasured confounding, despite the use of matching and multivariable adjustment. Sample size was modest, and the number of events limited precision in subgroup and matched analyses. The study period encompassed significant advances in PCI technique, stent platforms, and perioperative care, which may have introduced era effects, although stratified analyses did not reveal significant temporal heterogeneity. Procedural complications, particularly bleeding, may have been underestimated, and the LT selection process inherently excludes patients with prohibitive cardiac risk, further limiting generalizability.

Despite these limitations, this study has several strengths, including the exclusive inclusion of patients with angiographically confirmed severe CAD, consistent definitions of disease severity and outcomes, and the application of multiple analytic methods. Long-term follow-up provided robust estimates of event rates, which demonstrated that over half of patients experienced a MACE despite selective PCI. The consistent lack of association between PCI and improved outcomes across analytic approaches indicates that any potential benefit may be small. The identification of prior stroke, prior myocardial infarction, and dialysis dependence as independent predictors of adverse outcomes highlights the need for risk stratification strategies that extend beyond coronary anatomy alone.

These results indicate that PCI may not reduce post-transplant cardiovascular risk in asymptomatic LT candidates with severe CAD. While definitive conclusions require randomized controlled trials, these findings should be considered hypothesis-generating. Future prospective studies incorporating contemporary PCI strategies, standardized bleeding and ischemic endpoints, and stratification by coronary anatomy are required to clarify whether any subgroup of LT candidates benefits from prophylactic PCI. Until such data becomes available, revascularization decisions should be made on an individualized basis within a multidisciplinary framework, balancing procedural risk against the currently uncertain potential for benefit.

## 5. Conclusions

Among LT recipients with asymptomatic, angiographically severe CAD, PCI pre-LT was not associated with a reduction in post-LT MACE compared with medical therapy alone. These results are consistent with prior non-cardiac surgical studies and underscores the need for individualized, multidisciplinary decision-making. Prospective trials are necessary to guide evidence-based recommendations for the management of CAD in LT candidates.

## Figures and Tables

**Table 1:** Basic patient demographics of those who were revascularized.

Characteristics	Not Revascularized (N = 45 ^1^)	Revascularized (N = 66 ^1^)	*p*-Value ^2^
Gender			0.9
Male	38 (84%)	55 (83%)	
Female	17 (16%)	11 (17%)	
Race			0.12
White	36 (80%)	61 (92%)	
Asian	2 (4.4%)	3 (4.5%)	
Black	3 (6.7%)	1 (1.5%)	
Other	4 (8.9%)	1 (1.5%)	
Age at Transplant	64 (60, 70)	66 (58, 69)	>0.9
Obesity	28 (62%)	27 (41%)	0.027
MASLD	23 (51%)	44 (67%)	0.1
Alcohol Liver Failure	18 (40%)	14 (21%)	0.032
Hepatitis C	8 (18%)	10 (15%)	0.7
Hepatocellular Carcinoma	23 (51%)	32 (48%)	0.8
Hepatitis B	2 (4.4%)	6 (9.1%)	0.5
Alpha-1-antitrypsin	2 (4.4%)	3 (4.5%)	>0.9
Primary Sclerosing Cholangitis	1 (2.2%)	3 (4.5%)	0.6
Primary Biliary Cirrhosis	2 (4.4%)	0 (0%)	0.2
Autoimmune Hepatitis	1 (2.2%)	1 (1.5%)	>0.9
Cryptogenic Cirrhosis	11 (24%)	32 (48%)	0.011
Hemochromatosis	1 (2.2%)	3 4.%)	0.6
Other Need for Transplant	2 (4.4%)	6 9.1%)	0.5
MELD Pre-Transplant	28 (23, 30)	28 (23.5, 35)	0.6
Hypertension	6 (13%)	9 (14%)	>0.9
Hyperlipidemia	12 (27%)	26 (39%)	0.2
Diabetes	14 (31%)	26 (39%)	0.4
Previous Stroke	2 (4.4%)	4 (6.1%)	>0.9
Previous Myocardial Infarction	14 (31%)	18 (27%)	0.7
Previous or Current Smoker	12 (27%)	24 (36%)	0.3
Dialysis	5 (11%)	13 (20%)	0.2

^1^ n (%); Median (Q1, Q3), ^2^ Pearson’s Chi-squared test; Fisher’s exact test; Wilcoxon rank sum test.

**Table 2:** Distribution of Outcomes by Revascularization Status.

Outcomes	No Revascularization	Revascularization	*p*-Value
Overall MACE	27 (60%)	34 (52%)	0.40
Myocardial Infarction (MACE)	14 (31%)	12 (18%)	0.11
Stroke (MACE)	4 (8.9%)	3 (4.5%)	0.40
Revascularization after Transplant (MACE)	3 (6.7%)	4 (6.1%)	>0.9
Systolic Dysfunction (MACE)	16 (36%)	15 (23%)	0.14
Death (MACE)	8 (18%)	14 (21%)	0.70
Major Bleeding	2 (4%)	6 (9%)	0.47

**Table 3:** Hazard Ratio of Outcomes by Revascularization.

Outcomes	HR	CI	*p*-Value
Combined MACE	1.16	0.68, 1.96	0.6
Myocardial Infarction (MACE)	0.73	0.33, 1.61	0.4
Stroke (MACE)	0.5	0.11, 2.22	0.4
Revascularization (MACE)	0.9	0.20, 4.01	0.9
Systolic Dysfunction (MACE)	0.66	0.32, 1.35	0.3
Death (MACE)	1.73	0.68, 4.37	0.2
Major Bleeding	1.87	0.38, 9.34	0.4

**Table 4:** Risk Ratios, Attributable Risks, and Odds Ratios for Outcomes Based on Revascularization Status.

Outcomes	Number of Patients Revascularized	Number of Patients Not Revascularized	Risk Ratio (95% CI)	Attributable Risk (95% CI)	Odds Ratio (95% CI)
Overall MACE	34	27	0.86 (0.61–1.20)	−8.48 (−27.20–10.23)	0.71 (0.33–1.53)
Myocardial Infarction	12	14	0.58 (0.30–1.14)	−12.93 (−29.35–3.49)	0.49 (0.20–1.20)
Stroke	3	4	0.51 (0.12–2.18)	−4.34 (−14.06–5.37)	0.49 (0.10–2.29)
Revascularization	4	3	0.91 (0.21–3.87)	−0.61 (−9.89–8.68)	0.90 (0.19–4.24)
Systolic Dysfunction	15	16	0.64 (0.35–1.16)	−12.83 (−30.09–4.43)	0.53 (0.23–1.23)
Death	14	8	1.19 (0.55–2.61)	3.43 (−11.47–18.34)	1.25 (0.47–3.27)
Major Bleeding	6	2	2.05 (0.43–9.68)	4.65 (−4.54–13.83)	2.15 (0.41–11.17)

**Table 5:** Anatomic Locations of Severe Coronary Artery Disease by Revascularization Status.

Coronary Arteries	Not Revascularized	Revascularized	*p*-Value
Left Main	2	7	0.31
Left Anterior Descending	14	49	<0.01
Left Circumflex	2	10	0.12
Right Coronary	12	17	1
Branch Vessels	22	19	0.04

**Table 6:** Hazard Raio of MACE by Severe Coronary Artery Disease Revascularization.

**Coronary Arteries**	**MACE Composite**			**Myocardial Infarction**		
	**Number of Patients**	**HR (95% CI)**	***p*-Value**	**Number of Patients**	**HR (95% CI)**	***p*-Value**
Left Main	3	0.60 (0.19–1.91)	0.38	1	0.49 (0.07–3.60)	0.48
Left Anterior Descending	37	1.60 (0.94–2.71)	0.08	13	1.03 (0.47–2.29)	0.93
Left Circumflex	6	1.00 (0.43–2.33)	0.99	2	0.72 (0.17–3.07)	0.66
Right Coronary	16	0.84 (0.47–1.50)	0.55	9	1.17 (0.50–2.71)	0.72
Branch Vessels	27	1.32 (0.79–2.20)	0.28	12	1.48 (0.67–3.27)	0.33
**Coronary Arteries**	**Stroke/TIA**			**Revascularization**		
	**Number of Patients**	**HR (95% CI)**	***p*-Value**	**Number of Patients**	**HR (95% CI)**	***p*-Value**
Left Main	0	NA	1.0	0	NA	1.0
Left Anterior Descending	5	1.94 (0.38–9.99)	0.43	5	2.01 (0.39–10.36)	0.40
Left Circumflex	1	1.40 (0.17–11.60)	0.76	0	NA	1.0
Right Coronary	0	NA	1.0	2	1.08 (0.21–5.56)	0.93
Branch Vessels	2	0.69 (0.13–3.57)	0.66	4	2.50 (0.56–11.22)	0.23
**Coronary Arteries**	**Death**					
	**Number of Patients**	**HR (95% CI)**	***p*-Value**			
Left Main	2	1.33 (0.31–5.72)	0.70			
Left Anterior Descending	13	1.29 (0.54–3.08)	0.57			
Left Circumflex	3	1.60 (0.47–5.49)	0.45			
Right Coronary	7	1.26 (0.51–3.15)	0.62			
Branch Vessels	12	2.02 (0.86–4.77)	0.11			

NA = Not Applicable.

**Table 7:** Baseline Characteristics by Revascularization Status (Matched Cohort).

Characteristics	Not Revascularized (N = 13 ^1^)	Revascularized (N = 23 ^1^)	*p*-Value ^2^
Gender			0.33
Male	10 (77%)	21 (91%)	
Female	3 (23%)	2 (9%)	
Race			0.44
White	11 (85%)	20 (87%)	
Asian	0	2 (9%)	
Black	1 (8%)	0	
Other	1 (8%)	1 (4%)	
Age at Transplant	67 (59, 69)	64 (61, 70)	0.96
Obesity	9 (69%)	5 (22%)	0.01
MASLD	6 (46%)	13 (57%)	0.73
Alcohol Liver Failure	5 (38%)	6 (26%)	0.47
Hepatitis C	3 (23%)	8 (35%)	0.71
Hepatocellular Carcinoma	5 (38%)	13 (57%)	0.49
Hepatitis B	0	2 (9%)	NA
Alpha-1-antitrypsin	0	0	NA
Primary Sclerosing Cholangitis	0	1 (4%)	NA
Primary Biliary Cirrhosis	1 (8%)	0	NA
Autoimmune Hepatitis	1 (8%)	1 (4%)	1.00
Cryptogenic Cirrhosis	5 (38%)	10 (43%)	1.00
Hemochromatosis	0	2 (9%)	NA
Other Need for Transplant	0	2 (9%)	NA
MELD Pre-Transplant	28 (25, 30)	28 (25, 35)	0.97
Hypertension	3 (23%)	3 (13%)	0.65
Hyperlipidemia	4 (31%)	7 (30%)	1.00
Diabetes	5 (38%)	9 (39%)	1.00
Previous Stroke	0	0	NA
Previous Myocardial Infarction	2 (15%)	3 (13%)	1.00
Previous or Current Smoker	2 (15%)	10 (43%)	0.14
Dialysis	0	0	NA

^1^ n (%); Median (Q1, Q3), ^2^ Pearson’s Chi-squared test; Fisher’s exact test; Wilcoxon rank sum test; NA = Not Applicable

**Table 8:** Hazard Ratio of MACE by Revascularization (Matched Cohort).

MACE	No Revascularization (N = 13)	Revascularization (N = 23)	HR	CI	*p*-Value
Combined MACE	8 (62%)	8 (35%)	0.33	0.08, 1.33	0.13
Myocardial Infarction	3 (23%)	3 (13%)	0.50	0.08, 3.13	0.44
Stroke	1 (8%)	0 (0%)	NA	NA	1.00
Revascularization	1 (8%)	0 (0%)	NA	NA	1.00
Systolic Dysfunction	5 (38%)	6 (26%)	0.56	0.13, 2.48	0.44
Death	2 (15%)	3 (13%)	0.83	0.12, 6.99	0.85
Major Bleeding	1 (8%)	3 (13%)	NA	NA	1

NA = Not Applicable.

**Table 9:** Hazard Ratio of Outcomes by Revascularization in the 2005–2015 & 2016–2025 Eras.

**A: Hazard Ratio of Outcomes by Revascularization in the 2005–2015 Era**			
**Outcomes**	**HR**	**CI**	***p*-Value**
Combined MACE	1.16	0.40–3.41	0.78
Myocardial Infarction (MACE)	0.84	0.16–4.33	0.83
Stroke (MACE)	NA	NA	NA
Revascularization (MACE)	NA	NA	NA
Systolic Dysfunction (MACE)	0.75	0.15–3.71	0.72
Death (MACE)	2.41	0.53–10.84	0.25
Major Bleeding	2.53	0.36–18.00	0.35
**B: Hazard Ratio of Outcomes by Revascularization in the 2016–2025 Era**			
Outcomes	**HR**	**CI**	***p*-Value**
Combined MACE	0.82	0.44–1.53	0.54
Myocardial Infarction (MACE)	0.62	0.25–1.59	0.32
Stroke (MACE)	0.47	0.09–2.32	0.35
Revascularization (MACE)	0.94	0.17–5.12	0.94
Systolic Dysfunction (MACE)	0.60	0.26–1.38	0.23
Death (MACE)	1.21	0.38–3.87	0.74
Major Bleeding	1.47	0.15–14.16	0.74

NA = Not Applicable.

## Data Availability

Due to the sensitive nature of patient data, the datasets are not publicly available but may be obtained from the corresponding author with appropriate institutional approvals.

## Supplementary Materials

The following supporting information can be downloaded at: https://www.mdpi.com/article/10.3390/jcm14197067/s1, Supplementary Figure S1A. Probability of Myocardial Infarction by Revascularization Status, Supplementary Figure S1B. Probability of Stroke/TIA by Revascularization Status. Supplementary Figure S1C. Probability of Post Liver Transplant Revascularization by Revascularization Status. Supplementary Figure S1D. Probability of Post Liver Transplant Systolic Dysfunction by Revascularization Status. Supplementary Figure S1E. Probability of Death by Revascularization Status. Supplementary Figure S2. Love Plot showing covariate balance for matched cohort. Supplementary Table S1: Event Rates for MACE by Revascularization Status. Supplementary Table S2: Cohen’s D Effect Size.

## Abbreviations

The following abbreviations are used in this manuscript:

AASLD	American Association for the Study of Liver Disease
ACC	American College of Cardiology
AHA	American Heart Association
BARC	Bleeding Academic Research Consortium
CABG	Coronary artery bypass graft
CAD	Coronary Arter Disease
CCTA	Coronary Computed Tomographic Angiography
CEM	Coarsened exact matching
CR	Coronary Revascularization
DAPT	Dual Antiplatelet Therapy
EASL	European Association for the Study of the Liver
ECG	electrocardiogram
FFR	Fractional Flow Reserve
LAD	Left Anterior Descending Artery
LCx	Left Circumflex Artery
LT	Liver Transplantation
MACE	Major adverse cardiovascular events
MASLD	Metabolic dysfunction-associated steatotic liver disease
MI	Myocardial infarction
MRI	Magnetic Resonance Imaging
PCI	Percutaneous Coronary Intervention
RCA	Right Coronary Artery
sHR	Subdistribution hazard ration
TIA	Transient Ischemic Attacks
