# Supplementary material for: Outcomes in Asymptomatic Patients Undergoing Coronary Revascularization Before Liver Transplantation
Source: J Clin Med. 2025 Oct 7;14(19):7067. doi: 10.3390/jcm14197067 (PMC12525977; doi:10.3390/jcm14197067)
Supplement: Supplementary file 1 [file jcm-14-07067-s001.zip › jcm-3859094-supplementary.pdf]

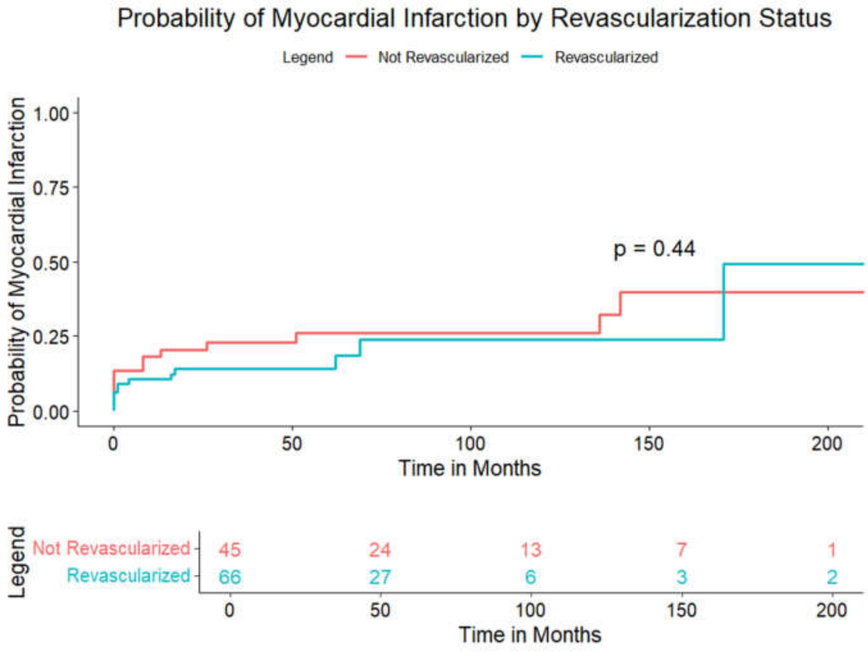

Supplementary Figure S1A. Probability of Myocardial Infarction by Revascularization Status.

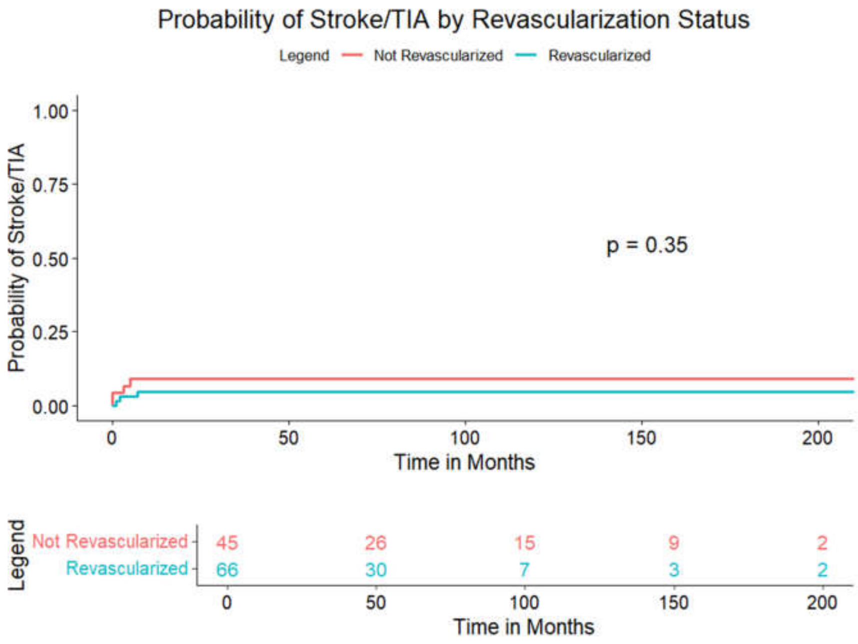

Supplementary Figure S1B. Probability of Stroke/TIA by Revascularization Status.

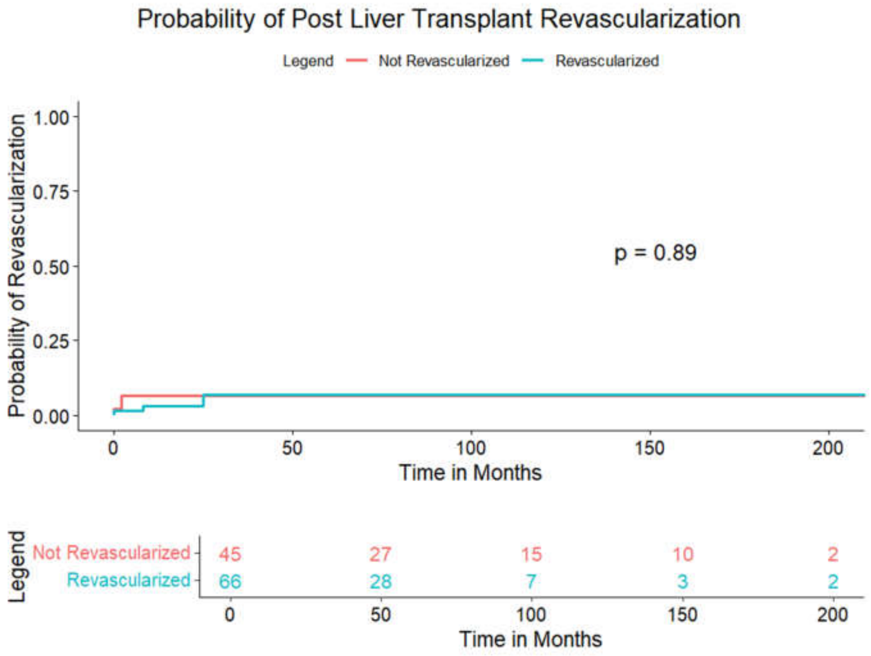

Supplementary Figure S1C. Probability of Post Liver Transplant Revascularization by Revascularization Status.

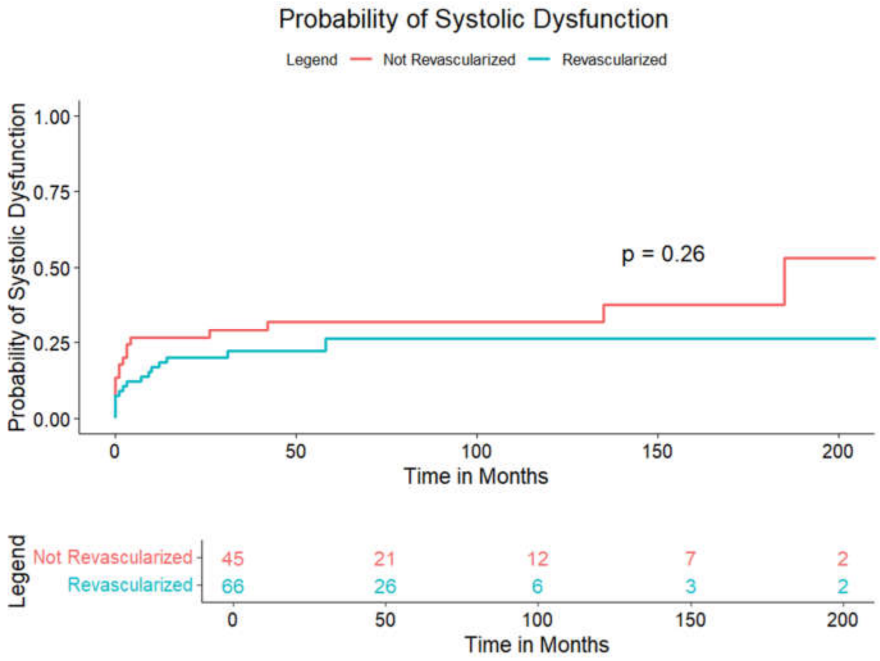

Supplementary Figure S1D. Probability of Post Liver Transplant Systolic Dysfunction by Revascularization Status.

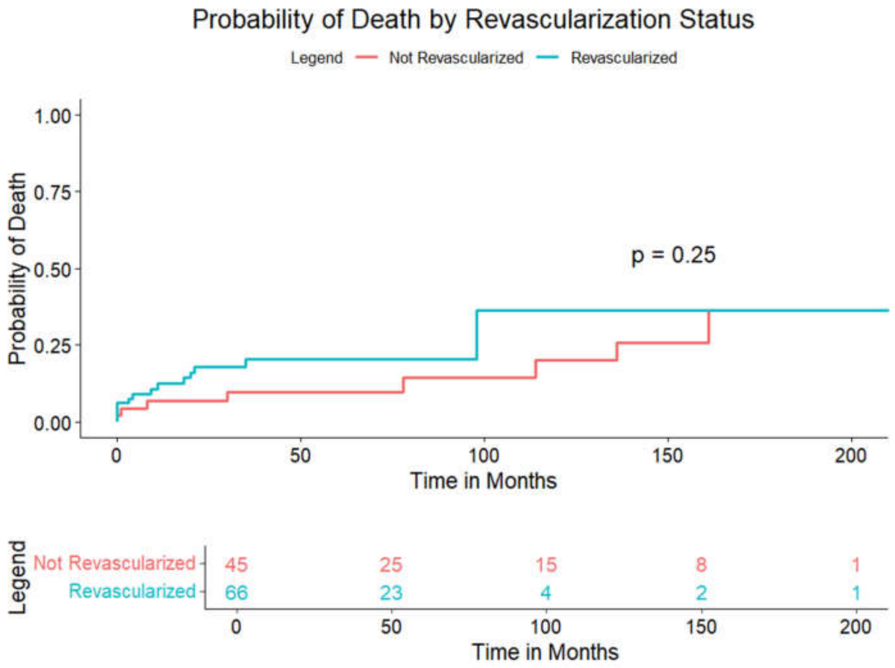

Supplementary Figure S1E. Probability of Death by Revascularization Status.

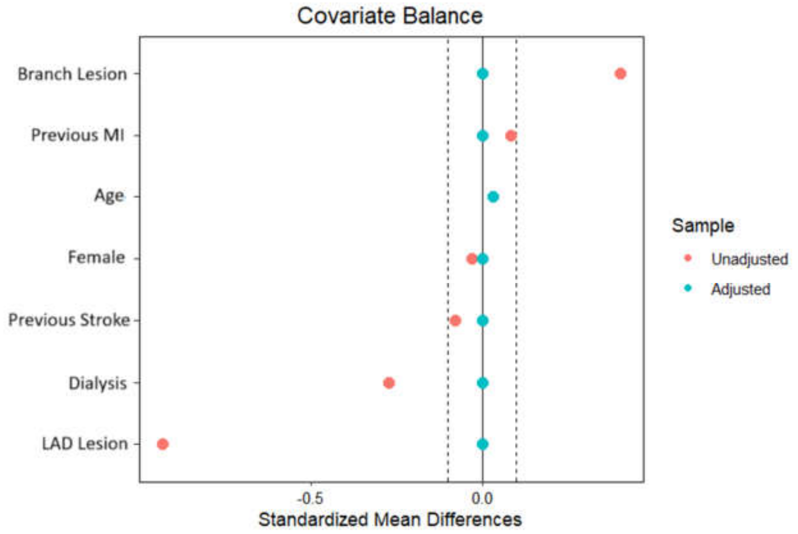

Supplementary Figure S2. Love Plot showing covariate balance for matched cohort.

Supplementary Table S1. Event Rates for MACE by Revascularization Status.

Supplementary Table 1: Event Rates for MACE by Revascularization Status				
**MACE**	**Revasculariza-tion Status**	**Events**	**Person Years**	**Rate Per 100 Person Years**
Overall MACE	Revascularized	34	223	15.2
Overall MACE	Not Revascular-ized	27	243	11.1
Myocardial In-farction	Revascularized	12	277	4.33
Myocardial In-farction	Not Revascular-ized	14	272	5.14
Stroke	Revascularized	3	305	0.98
Stroke	Not Revascular-ized	4	306	1.31
Revasculariza-tion	Revascularized	4	295	1.35
Revasculariza-tion	Not Revascular-ized	3	313	0.96
Systolic Dys-function	Revascularized	15	267	5.62
Systolic Dys-function	Not Revascular-ized	16	257	6.23
Death	Revascularized	14	246	5.69
Death	Not Revascular-ized	8	296	2.70
Major Bleeding	Revascularized	60	498	12.00
Major Bleeding	Not Revascular-ized	43	84	51.00

SupplementaryTable S2. Cohen's D Effect Size.

Supplementary Table 2: Cohen's D Effect Size for the Effect of Revascularization on MACE		
**MACE**	**Cohen's D**	**CI**
Overall MACE	0.17	-0.21, 0.55
Myocardial Infarction	0.31	-0.08, 0.69
Stroke	0.18	-0.20, 0.56
Revascularization	0.02	-0.35, 0.40
Systolic Dysfunction	0.29	-0.10, 0.67
Death	-0.09	-0.46, 0.29
